# Supplementary material for: A peripheral epigenetic signature of immune system genes is linked to neocortical thickness and memory
Source: Nat Commun. 2017 Apr 26;8:15193. doi: 10.1038/ncomms15193 (PMC5414038; doi:10.1038/ncomms15193)
Supplement: Supplementary Information — Supplementary Figures, Supplementary Tables, Supplementary Notes and Supplementary References [file ncomms15193-s1.pdf]

Supplementary Figures

Supplementary Figure 1: Distribution of *ICA2*-contributing CpGs based on topographical distribution **(A)** and relative location to gene transcript **(B)**.

Background corresponds to the genome-wide distribution of CpGs across the arrays. *ICA2* distribution was compared to background distributions using goodness of fit χ^2 test: *p*: p-value; stars indicates the magnitude of the bins standardized residuals: *: > |2|, **: >|3|, ***: > |4|.

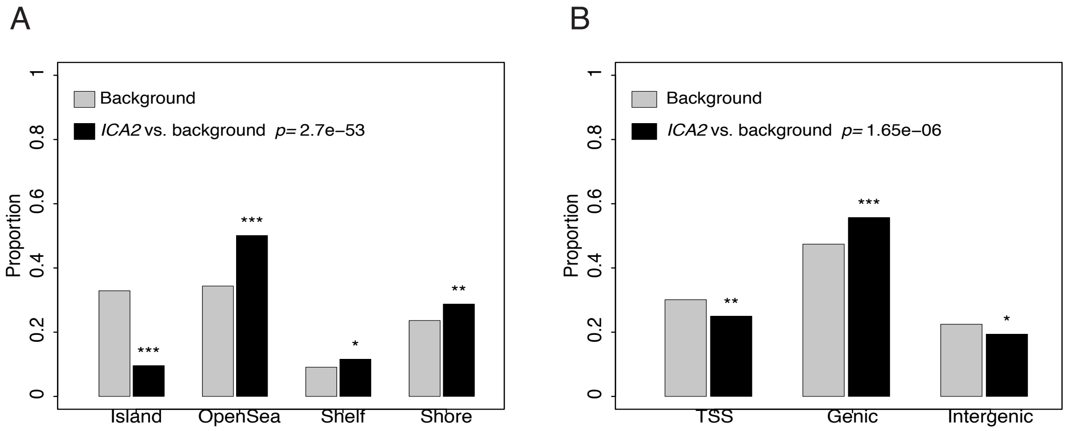

Supplementary Figure 2: Q-Q plot of mQTL analysis between 71 GSEA genetic score SNPs and *ICA2* CpGs.

Red line shows expected uniform distribution.
Blue dashed line indicates the 95 % quantiles obtained from 1000 repeats of association testing between *ICA2* CpGs and randomly selected cis-SNPs.

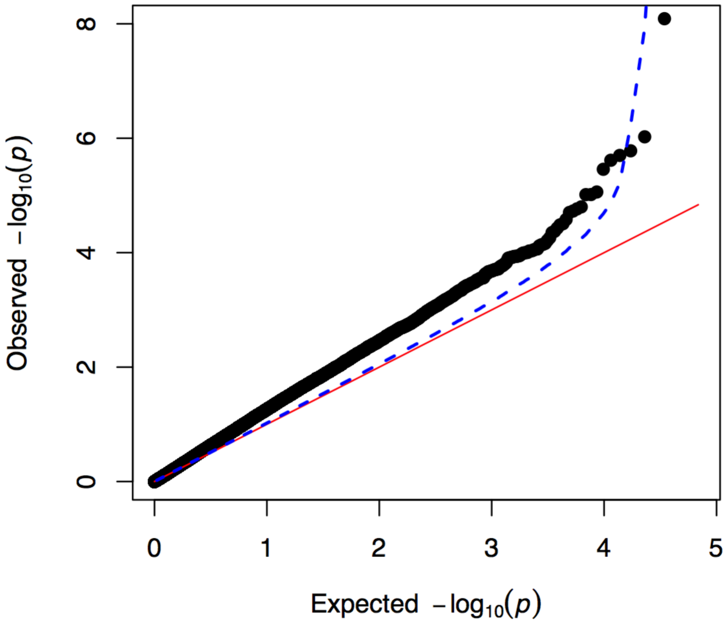

Supplementary Figure 3: Average whole-blood DNAm at 970 *ICA2* CpGs versus progenitor cell specific DNAm.

Horizontal axis: average whole-blood DNAm observed in the methylomic Swiss sample (n=533).

Vertical axis: average DNAm observed in progenitor cells.

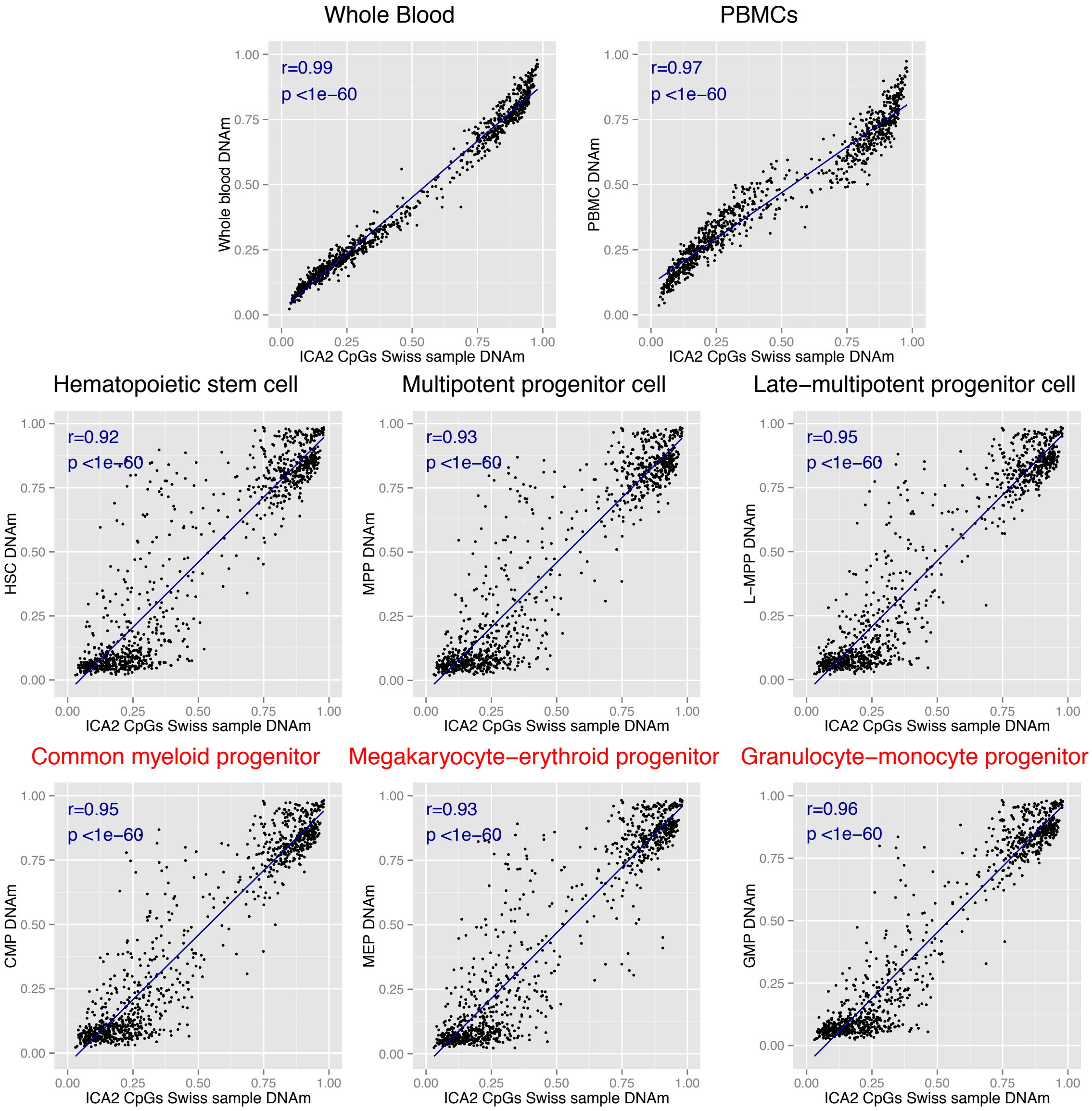

Supplementary Figure 4: Average whole-blood DNAm at 970 *ICA2* CpGs versus cell subtypes specific DNAm.

Horizontal axis: average whole-blood DNAm observed in the methylomic Swiss sample (n=533).

Vertical axis: average DNAm observed in specific cell subtypes.

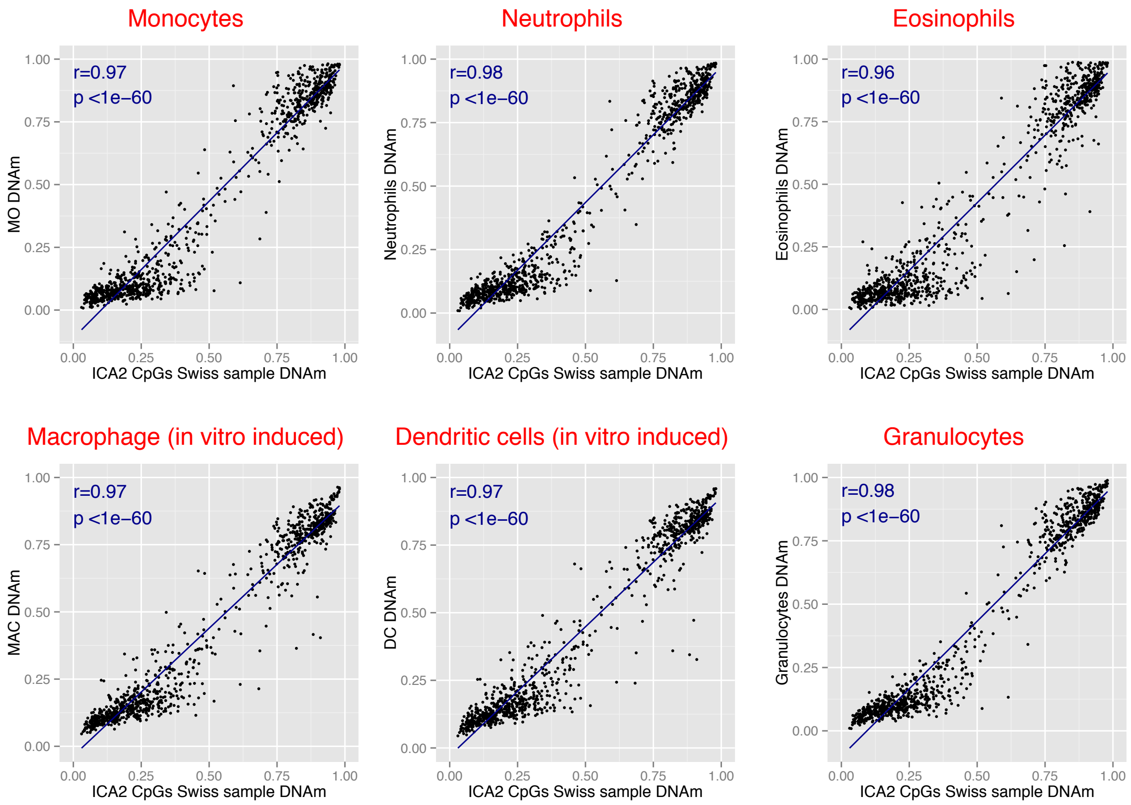

Supplementary Figure 5: Average whole-blood DNAm at 970 *ICA2* CpGs versus lymphocytes subtypes specific DNAm.

Horizontal axis: average whole-blood DNAm observed in the methylomic Swiss sample (n=533).

Vertical axis: average DNAm observed in specific cell subtypes.

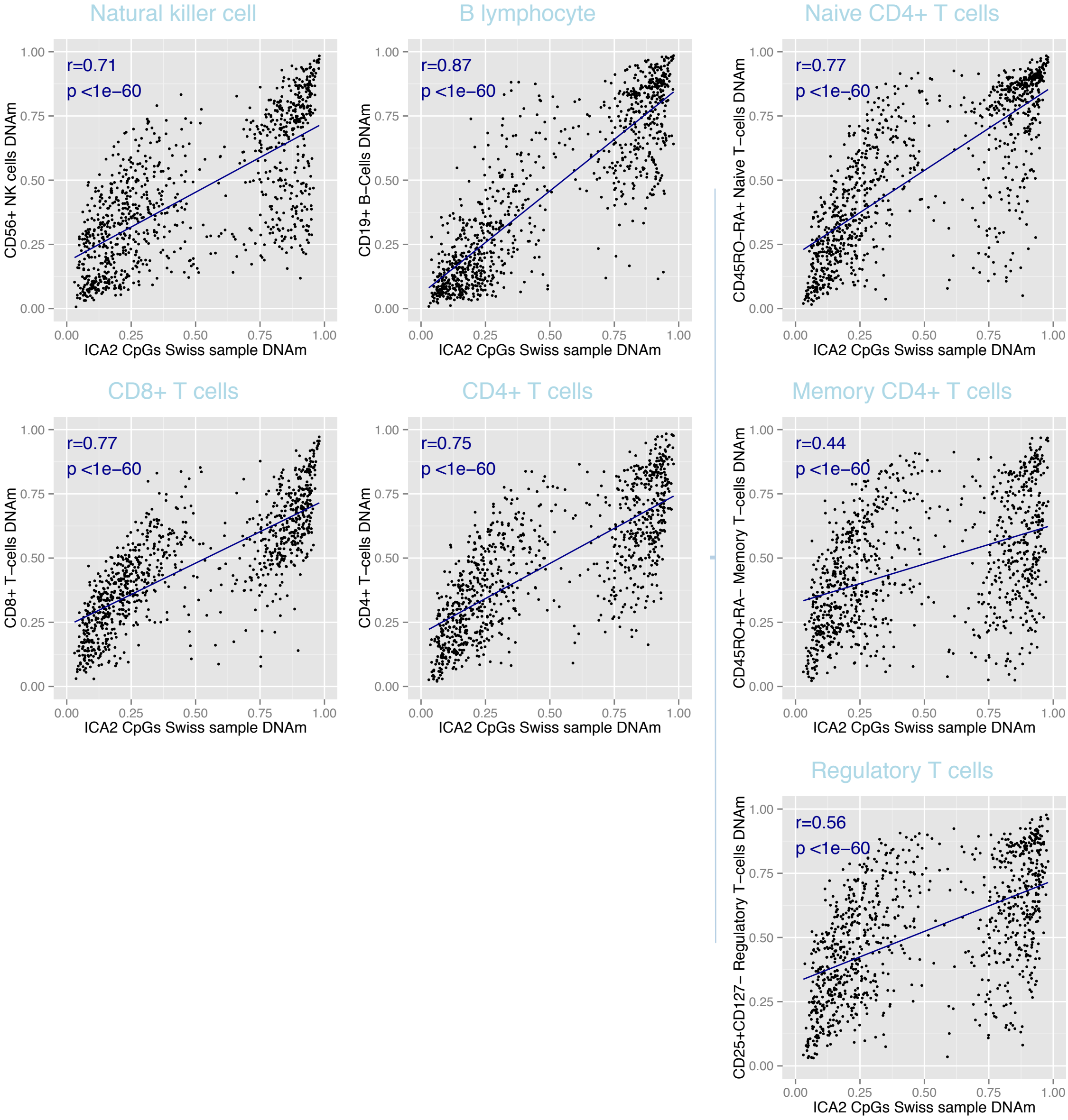

Supplementary Tables

Supplementary Table 1: Description of methylomic profiling and imaging analysis samples.

	Methylomic profiling sample	Basel imaging sample
N	533	722
Age at MRI/behavioral assessment in years (mean ± SD)	22.9 ± 3.3	22.9 ± 3.3
Age blood sampling in years (*)	23.9 ±3.5	/
Number of females (%)	311 (58.3%)	447 (61.9%)
Episodic Memory-*N*	531	722
Structural imaging-*N* (including Episodic Memory-*N)*	514 (512)	722 (722)

(*): blood sampling for methylomic profiling.

Supplementary Table 2: Correlations between 15 ICA methylomic patterns and global thickness.

(a) Cortical measures were adjusted for sex, intracranial volume and MR-technical batches using linear regression. (b) Chronological age effects were further partialled out from cortical measures and *ICA* component. r: Pearson's correlation coefficient; *p*: two-sided test p-value.

	**Thickness** ***N = 514***				**Age** ***N = 533***	
	**No age adjustment**		**Age adjustment**			
**ICA**	**r**	***p***	**r**	***p***	**r**	***p***
ICA1	-0.14	0.00162	0.01	0.83	0.54	1.54E-42
ICA2	-0.24	3.86E-08	-0.18	6.55E-05	0.29	4.68E-12
ICA3	-0.03	0.548	-0.01	0.901	0.08	0.0578
ICA4	0.01	0.837	-0.01	0.806	-0.08	0.0691
ICA5	-0.06	0.166	-0.08	0.0721	-0.06	0.166
ICA6	0.1	0.0282	0.08	0.0545	-0.06	0.166
ICA7	0.01	0.819	0	0.937	-0.05	0.281
ICA8	0.04	0.414	0.04	0.396	-0.04	0.337
ICA9	0.09	0.0368	0.08	0.0585	-0.04	0.339
ICA10	-0.03	0.467	-0.03	0.566	0.03	0.42
ICA11	0.03	0.465	0.04	0.311	0.03	0.454
ICA12	-0.05	0.224	-0.06	0.153	-0.02	0.608
ICA13	-0.06	0.171	-0.06	0.148	-0.02	0.647
ICA14	0	0.992	0.01	0.885	0.02	0.68
ICA15	-0.04	0.369	-0.04	0.339	-0.02	0.695

Supplementary Table 3: Correlations between cortical thickness factor scores and *ICA2*.

r: Pearson's correlation coefficient. *p*: two-sided correlation test p-value.

**Factor**	***ICA2*** ***N = 514***	
	**r**	***p***
F1	-0.112	0.0108
F2	-0.09	0.0406
F3	-0.065	0.142
F4	-0.08	0.0707
F5	-0.013	0.774
F6	-0.13	0.00314
F7	0.004	0.935
F8	-0.013	0.776

Supplementary Table 4: Correlations between 15 ICA methylomic components and EM performance.

Age adjustment: chronological age effects were partialled out from each measure.
r: Pearson's correlation coefficient. *p*: two-sided correlation test p-value.

**ICA**	**Methylomic profiling sample** ***N= 531***			
	**No Age adjustment**		**Age adjustment**	
	r	***p***	***r***	***p***
ICA1	-0.043	0.328	-0.003	0.944
ICA2	-0.138	0.00147	-0.122	0.00491
ICA3	0.011	0.809	0.017	0.696
ICA4	-0.018	0.683	-0.024	0.586
ICA5	0.021	0.627	0.017	0.699
ICA6	0.06	0.165	0.057	0.19
ICA7	-0.007	0.875	-0.009	0.829
ICA8	0.09	0.0375	0.089	0.0404
ICA9	-0.002	0.972	-0.005	0.909
ICA10	-0.015	0.739	-0.012	0.785
ICA11	-0.068	0.119	-0.066	0.13
ICA12	-0.011	0.804	-0.013	0.763
ICA13	0.017	0.691	0.016	0.711
ICA14	-0.023	0.598	-0.021	0.622
ICA15	0.039	0.364	0.039	0.374

Supplementary Table 5: Correlations between cortical thickness factor scores and EM performance.

r: Pearson's r correlation coefficient; *p*: two-sided correlation test p-value.

Factor	Combined sample (*N*=1234)		Basel imaging sample (*N*=722)		Methylomic profiling sample (*N*=512)	
	**r**	***p***	**r**	***p***	**r**	***p***
F1	-0.054	0.0575	-0.086	0.0215	-0.01	0.823
F2	-0.045	0.115	-0.072	0.0548	-0.006	0.901
F3	-0.012	0.663	-0.023	0.541	0.001	0.973
F4	-0.024	0.395	0.006	0.869	-0.071	0.107
F5	-0.05	0.0812	-0.04	0.288	-0.066	0.139
F6	0.079	0.00574	0.102	0.00617	0.048	0.283
F7	-0.007	0.814	-0.018	0.634	0.008	0.85
F8	0.026	0.361	0.05	0.183	-0.009	0.839

Supplementary Table 6: Association between age, global cortical thickness and EM performance and WBC counts.

Results from linear models analysis with phenotype as dependent variable and WBC counts as explanatory variables. *t*: t-statistic value; *F*: Overall effect F-statistic value; *p*: p-value
(a): Adjustment for sex, intra-cranial volume, MR batches and chronological age.
(b): Adjustment for sex and chronological age.
(c): Basophils, Eosinophils and Monocytes.

Phenotype	N		*t*	*p*
Age	527	Lymphocytes	0.84	0.40
		Neutrophils	0.31	0.76
		Mixture (c)	0.57	0.57
		$F(3,523) = 0.60$, $p = 0.62$		
Global cortical thickness (a)	509	Lymphocytes	-0.87	0.39
		Neutrophils	-2.32	0.02
		Mixture (c)	0.31	0.75
		$F(3,505) = 2.2$, $p = 0.087$		
EM performance (b)	525	Lymphocytes	1.69	0.093
		Neutrophils	0.21	0.84
		Mixture (c)	1.1	0.29
		$F(3,521) = 2.1$, $p = 0.10$		
Cortical thickness F6	509	Lymphocytes	0.65	0.51
		Neutrophils	-0.66	0.51
		Mixture (c)	-0.28	0.78
		$F(3,505) = 0.28$, $p = 0.84$		

Supplementary Notes

1. Description of Munich sample

1.1 Structural imaging

MRI acquisition: High resolution T1-weighted images were acquired at the Neuro-imaging Core Unit of the MPIP on a clinical 1.5 Tesla MR scanner (Signa/Signa Excite, General Electric, for sequence details see [1,2]). *MRI data processing:* Gross morphological abnormalities such as tumor or territorial infarction, ventricle asymmetries or arachnoid cysts preventing automated image processing, extensive white matter disease or motion artefacts were exclusion criteria prior to the formation of this combined sample. The surface-based segmentation stream of FreeSurfer (version 5.3, installed on 64-bit Linux workstations) was applied to all T1-weighted images, with substeps as described in the Structural Imaging section. Visual QC of cortical segmentation quality was performed on the basis of standardized protocols (http://enigma.ini.usc.edu/protocols/imaging-protocols) and led to exclusion of 12 subjects. As phenotypes of interest, left and right cortical thickness (the average of which is ref. to as cortical thickess [CT]), and intracranial volume derived indirectly from the spatial registration procedure.

1.2 Methylomic profiling

DNA was extracted from whole blood using the Gentra Puregene Blood Kit (QIAGEN). Quality and quantity of the DNA were assessed by NanoDrop 2000 Spectrophotometer (Thermo Scientific) and Quant-iT Picogreen (Invitrogen). Genomic DNA was bisulfite converted using the Zymo EZ-96 DNA Methylation Kit (Zymo Research) and genome-wide methylome levels were assessed with the Illumina Infimium HumanMethylation 450K BeadChip array. Hybridization and processing was performed according to manufacturer' s instructions. Intensity read outs, normalization and estimation and

beta values were obtained using the Minfi package (version 1.21.0) in Bioconductor [3]. Beta values for the pre-selected 397,947 autosomal probes from the Swiss sample were calculated from SWAN normalized intensities. After pre-processing of methylomic data, and MRI-QC based exclusions, combined data of N=596 subjects was available for statistical analysis.

2. Description of AgeCode sample

Briefly, participants were recruited between January 2003 and November 2004 in six German study centers (Bonn, Düsseldorf, Hamburg, Leipzig, Mannheim, Munich) via general practitioners (GP) connected to the respective study sites. Inclusion criteria were age of 75 years and older, absence of dementia (according to the GP's judgment) and at least one contact with the GP within the last 12 months. Exclusion criteria were GP consultations by home visits only, residence in a nursing home, presence of a severe illness with an anticipated fatal outcome within three months, insufficient German language abilities, deafness or blindness, lack of ability to provide an informed consent and status as being only an occasional patient of the participating GP. A total of 3'327 subjects were successfully contacted and assessed with structured clinical interviews at their homes. A total of 110 individuals were excluded after the first interview due to presence of dementia or an actual age below 75 (falsely classified as 75 or older in the sample selection process). For the present analyses, data from baseline and three follow-up measurements with 18 months intervals were available. In a primary care-based sample of older individuals, conditions can be present that affect cognition and the reliability of neuropsychological tests. In order to generate a sample of healthy elderly individuals we further employed the following selection criteria at baseline: Age between 75 and 90 years, German as native language, at least school-leaving certificate, absence of severe hearing or vision impairments, absence of insufficient test motivation

as judged by the interviewer, absence of disturbing factors during neuropsychological testing and absence of all of the following comorbid conditions: Parkinson's disease, epilepsy, alcohol abuse, stroke, multiple sclerosis, evidence of depression (a score of 6 or higher on the Geriatric Depression Scale [4]), traumatic brain injury with unconsciousness of more than 30 minutes, visible neurological malfunctions and dementia according to DSM-IV criteria [5]. In addition, we excluded subjects who converted to dementia up to the third follow-up or without neuropsychological test data available on baseline and all follow-up visits. After application of these selection criteria, a total of 1244 subjects remained in the sample. Sufficient DNA-samples for genome-wide genotyping were available for 782 subjects.

3. Description Episodic Memory phenotypes

3.1 Methylomic and Basel imaging samples

While undergoing fMRI acquisition, all participants completed a picture delayed free recall task. Stimuli consisted of 72 emotional and neutral pictures (24 negative, 24 positive and 24 neutral) taken from the International Affective Picture System (IAPS) [6] and from in-house standardized picture sets. Four additional pictures showing neutral objects were used to control for primacy and recency effects in memory. These pictures were not included in the analysis. Additionally, 24 scrambled pictures were included. Their background contained the color information of all pictures used in the experiment and was overlaid with a crystal and distortion filter (Adobe Photoshop CS3, Adobe Systems Inc., San Jose, CA, USA). On the foreground geometrical figures of varying shape, size and orientation were shown. Pictures were presented for 2.5 s each in a quasi-randomized order so that a maximum of four pictures of the same category (e.g. animals, humans, landscape) and valence occurred consecutively. Between the pictures a fixation-cross appeared on the screen for 500ms and the trials were separated by a

variable intertrial period of 9-12 s. During this time subjects were asked to rate the presented picture for valence (negative, neutral, positive) and arousal (large, medium, small) on a three-point rating scales (Self Assessment Manikin). Scrambled pictures were rated according to their shape (vertical, symmetric or horizontal) and size (large, medium, small). Subjects were not instructed to recall the pictures later (incidental recall). The delayed free recall was performed outside of the scanner, 10 min after presentation of all photographs. To document performance for the delayed recall of positive, negative, and neutral pictures, subjects had to describe each picture by writing it down in a few words. A picture was judged as correctly recalled if the rater could identify the presented picture based on the subject's description. Two blinded investigators independently rated the descriptions for recall success (inter-rater reliability > 99%). For the pictures, which were judged differently by the two raters (i.e. a particular picture was judged as correctly recalled by one rater but not the other), a third independent and blinded rater made a final decision with regard to whether the particular picture could be considered as successfully recalled. The number of correctly recalled pictures served as a phenotype. For initial association testing with *ICA2* pattern, EM performance was adjusted for sex effects using linear regression. For additional analyses, including genetic scoring analyses, EM performance was further adjusted for chronological age effect.

3.2 Basel cognitive sample

Participants performed the same pictures free recall task as described for the methylomic profiling sample, without fMRI assessment. EM performance, used in genetic scoring analysis, was adjusted for sex and chronological age effects using linear regression.

3.3 Zurich sample

Subjects viewed six series of five semantically unrelated nouns presented at a rate of one word per second with the instruction to learn the words for immediate free recall after each series. In addition, subjects underwent an unexpected delayed free-recall test of the learned words after 5min (episodic memory). The number of correctly recalled words (hits) was the relevant output. EM performance, used in genetic scoring analysis, was adjusted for sex and chronological age effects using linear regression.

3.4 AgeCode

Delayed recall performance as quantified by the Consortium to Establish a Registry for Alzheimer's Disease (CERAD) battery [7] served as phenotype. Subjects were presented a list of 10 words three times (presentation per word: 2 seconds), each time presented in a different order. After each run, subjects freely recalled as many words as possible. The number of correctly remembered items (free recall) after a 10 min delay served as the phenotypic measure. EM performance, used in genetic scoring analysis, was adjusted for sex and chronological age effects using linear regression.

4. Analysis of *ICA* methylomic patterns

4.1 Comparison of whole-blood and cell-specific DNAm values

Average DNA methylation values were obtained from four publically available datasets from 19 cell types. Average DNAm values from hematopoietic stem cells and progenitor cells were obtained from GSE63409 [8] considering only normal bone marrow samples. Average DNAm from whole-blood, PBMCs, Natural Killer cells, B-lymphocytes, CD4 T-cells, CD8 T-cells, monocytes, neutrophils, eosinophils and granulocytes were obtained from GSE3560 [9]. Average DNAm from specific sub-types of CD4 T-cells (naive, memory and regulatory CD4 T-cells) were obtained from GSE59250 [10] considering control

samples only. Average DNAm in dendritic cells and macrophage (in vitro induced) were obtained from GSE75937 [11].

4.2 ICA analysis of publically available whole-blood methylomic profiles

We analyzed whole-blood methylomic profiles from 656 samples reported in Hannum et al., 2013 [12]. In analogy to our methylomic dataset, multi-mapping or polymorphic probes were excluded from analysis. Raw intensities (methylated and unmethylated signals) were normalized using the lumi package (color-bias adjustment and quantile normalization). The BMIQ algorithm was finally applied to adjust for the difference between Type I and Type II probes used in the 450K array. Given substantial non-randomness of between-plate distribution of chronological age in this sample, we performed CoMbat adjustment for plate effect. DNA methylation values were subsequently adjusted for sex and 98 surrogate variables inferred from surrogate variable analysis (SVA). ICA decomposition on the adjusted signals yielded a total of 175 components, among which 19 were retained based on the per-subject 10% variance criterion used in our methylomic dataset. The retained ICA patterns were tested for association with age, after adjustment for estimated cell counts (CD4T, CD8T, NK, Gran, Mono, Bcell). Five patterns were significantly associated with age. In analogy to our study, CpGs contributing to these patterns were chosen so as to exhibit an absolute loading > |4| on the respective pattern.

4.3 Association of *ICA* patterns with chronological age in cell-specific methylomic profiles

We used publically available methylomic profiles from N=1202 monocytes samples (GSE56046) and N=214 CD4 T-cells samples (GSE56581) [13]. Normalized datasets deposited on GEO repository were considered for analysis. In each dataset a Surrogate Variable Analysis preserving for chronological age was performed. Individual methylomic values were adjusted for the inferred SVs using linear regression. In each

dataset, *ICA1* and *ICA2* patterns were estimated as the linear combination between the inverse of genome-wide *ICA1* and *ICA2* loadings (inferred from the Swiss sample) and scaled SV-adjusted DNAm values. This score was subsequently tested for association with chronological age.

5. mQTL analysis

5.1 Testing over-representation of genetic score SNPs in -cis to ICA2 CpGs

We randomly selected an equal number of SNPs from genome-wide genotyped SNPs and assessed the occurence of SNPs found in -*cis* (± 1 Mbp) to *ICA2* CpGs. This sampling procedure was repeated 5000 times to establish the null distribution and calculate the corresponding *p*-value.

5.2 Null distribution of cis-mQTL association statistics

First we determined all SNPs located within ± 1Mbp of any of the *ICA2* CpGs ('cis-SNP pool'). Association statistics were computed between *ICA2* CpGs and *n* SNPs randomly selected from the cis-SNP pool, with *n* equal to the number of GSEA genetic score SNPs (i.e. 71 SNPs), thus providing one realization of the baseline quantile distribution. This sampling procedure was repeated 1000 times.

Supplementary References

1. Stein, J. *et al.* Identification of common variants associated with human hippocampal and intracranial volumes. *Nat. Genet.* **44,** 552–61 (2012).

2. Hibar, D. P. *et al.* Common genetic variants influence human subcortical brain structures. *Nature* **520,** 224–9 (2015).

3. Aryee, M. J. *et al.* Minfi: a flexible and comprehensive Bioconductor package for the analysis of Infinium DNA methylation microarrays. *Bioinformatics* **30,** 1363–1369 (2014).

4. Yesavage, J. A. *et al.* Development and validation of a geriatric depression screening scale: a preliminary report. *J. Psychiatr. Res.* **17,** 37–49 (1983).

5. American Psychiatric Association. *Diagnostical and Statistical Manual of Mental Disorders (4th ed., text rev.).* (2000).

6. Lang, P. J., Bradley, M. & Cuthberth, B. N. International Affective Pictures System (IAPS): Affective Ratings of Pictures and Instruction Manual. *Univ. Florida, Gainesville, FL* (2008).

7. Welsh, K. A. *et al.* The consortium to establish a registry for Alzheimer's disease (CERAD). Part V. A normative study of the neuropsychological battery. *Neurology* **44,** 609–14 (1994).

8. Jung, N., Dai, B., Gentles, A. J., Majeti, R. & Feinberg, A. P. An LSC epigenetic signature is largely mutation independent and implicates the HOXA cluster in AML pathogenesis. *Nat. Commun.* **6,** 8489 (2015).

9. Reinius, L. *et al.* Differential DNA Methylation in Purified Human Blood Cells : Implications for Cell Lineage and Studies on Disease Susceptibility. *PLoS One* **7,** e41361 doi: 10.1371/journal.pone.0041361 (2012).

10. Absher, D. M. *et al.* Genome-Wide DNA methylation analysis of systemic lupus erythematosus reveals persistent hypomethylation of interferon genes and compositional changes to CD4+ T-cell populations. *PLoS Genet.* **9,** e1003678 doi: 10.1371/journal.pgen.1003678 (2013).

11. Vento-Tormo, R. *et al.* IL-4 orchestrates STAT6-mediated DNA demethylation leading to dendritic cell differentiation. *Genome Biol.* **17,** 4 doi: 10.1186/s13059–015–0863–2 (2016).

12. Hannum, G. *et al.* Genome-wide methylation profiles reveal quantitative views of human aging rates. *Mol. Cell* **49,** 359–67 (2013).

13. Reynolds, L. M. *et al.* Age-related variations in the methylome associated with gene expression in human monocytes and T cells. *Nat. Commun.* **5,** 5366 (2014).
